# Molecular pattern of acquiring upper respiratory infection

**DOI:** 10.3389/fmed.2026.1768374

**Published:** 2026-02-06

**Authors:** Mohamed A. Hendaus

**Affiliations:** 1 The View Hospital, in Affiliation with Cedar’s Sinai, Doha, Qatar; 2 Qatar University College of Medicine, Qatar University, Doha, Qatar

**Keywords:** cold, extracellular vesicle swarms, immunity, upper respiratory infection, winter

## Abstract

Upper respiratory tract infections (URIs) are a significant public health concern. Human behavior plays a crucial role in how often infected individuals come into contact with susceptible individuals. Among the key factors influencing the seasonality of these infections, variations in temperature and absolute humidity are critical elements driving the increase in respiratory virus infections, particularly during the winter months. Traditionally, it has been believed that cold and flu viruses thrive in winter mainly because falling temperatures lead to people spending more time indoors, which facilitates easier transmission. However, compelling evidence suggests that biological factors also contribute to the increased risk of illness in colder weather. Recent studies indicate that active mucosal defenses against bacteria extend into the mucus itself, aided by the epithelial release of antimicrobial extracellular vesicle (EV) swarms. Unfortunately, these studies have also shown that EVs are significantly weakened by exposure to cold environments.

## 1 Cold and upper respiratory tract infections

Upper respiratory tract infections (URIs) constitute a pressing public health concern, significantly impacting productivity and leading to increased absenteeism in both workplaces and schools, while also placing considerable strain on the healthcare system (1).

URIs are not just minor inconveniences; they can result in a host of serious complications such as otitis media, sinusitis, bronchiolitis, pneumonia, and exacerbations of asthma or chronic obstructive pulmonary disease (COPD) (2). The viruses behind URIs exhibit remarkable evolutionary dynamism, characterized by high mutation rates that enable them to evade previously established immunity. This ongoing threat underscores the risk of unpredictable virus strains emerging and re-emerging, potentially triggering life-threatening epidemics and pandemics (3, 4).

Environmental factors play a crucial role in shaping host susceptibility, influencing airway defense mechanisms, and determining the viability and transmission of respiratory viruses. Human behavior significantly impacts how often infected individuals encounter those who are susceptible. Among the key factors influencing seasonality, variations in temperature and absolute humidity have emerged as critical elements driving the seasonal uptick in respiratory virus infections, particularly evident during winter months (5).

Conventional wisdom has long held that cold and flu viruses flourish in winter largely because dropping temperatures drive people indoors, facilitating easier transmission. However, compelling evidence suggests that biological factors also contribute to our heightened risk of illness in colder weather. As winter advances and temperatures plummet, the air typically dries out. For those suffering from asthma, COPD, or bronchitis, this dry air can trigger a cascade of symptoms—throat irritation, wheezing, coughing, and shortness of breath. The connection between cold weather and respiratory disorders can be attributed to several critical factors (6). At the heart of the issue is the impact of dry air. Our airways are naturally bathed in a thin layer of fluid. When we inhale dry air, this fluid evaporates rapidly—sometimes faster than it can be replenished—resulting in throat dryness that leads to irritation and swelling, ultimately intensifying the symptoms of COPD and asthma. Moreover, cold weather prompts an uptick in mucus production. While mucus serves as a protective barrier for the throat, the mucus generated in cold conditions tends to be thicker and stickier than usual. This can result in blockages within the respiratory system, thereby elevating the likelihood of contracting colds or other infections (7).

Could the heightened risk of illness during colder weather be linked to the way low temperatures diminish the efficacy of antimicrobial extracellular vesicle (EV) swarms in our mucus, which serve as a crucial line of defense?

## 2 Nasal cavity

The nasal cavity stands as one of the first lines of defense between the external environment and the human body, demonstrating remarkable sensitivity to shifts in ambient temperature (8). The nasal mucosal barrier is therefore crucial in safeguarding against inhaled respiratory pathogens, employing multiple immune mechanisms. The physical barrier created by the nasal mucosa works tirelessly to prevent pathogens from entering the body through the production of mucus glycoproteins, mucociliary clearance, and tight junctions among epithelial cells (9). Furthermore, nasal epithelial cells play vital roles in initiating, maintaining, and regulating innate immunity. These defense mechanisms are inherently active and can be stimulated by both membrane-bound and cytoplasmic pattern recognition receptors that detect pathogen-associated molecular patterns typically present in viruses (10). Exposure to low temperatures triggers significant physiological changes in both humans and animals, leading to important cellular and molecular adaptations that serve as defenses against cold-induced damage. Cold environments increase mucus viscosity and impair ciliary action in the upper respiratory system, resulting in reduced clearance of pathogens (11).

## 3 Response of the human body to cold temperatures

When temperatures drop below 0 °C, the integrity of physical barriers is compromised, and immune functions become impaired, heightening the risk of infections from various pathogens. Furthermore, cold exposure leads to a marked decrease in lymphoproliferation while significantly elevating levels of stress hormones such as corticosteroids, catecholamines, epinephrine, norepinephrine, cortisol, and aldosterone (12). This heightened hormonal state results in leukocytosis and suppresses the production of inflammatory factors and adhesion molecules, fundamentally altering immune responses (13).

Cold stress profoundly impacts both the innate and adaptive immune systems. When the body encounters suboptimal temperatures, it strategically reallocates energy to prioritize heat generation, which drastically reduces the energy available to the immune system (14). Furthermore, cold stress has a markedly suppressive effect on leukocyte cellularity in key immune organs, including the blood, kidneys, lymph nodes, and spleen. This suppression occurs largely due to the inhibition of T and B lymphocyte proliferation and activation (15). Moreover, exposure to low temperatures can significantly decrease the levels of plasma proteins and enzymes essential for critical functions like membrane transport and skeletal mineralization. Research reveals that cold stress not only diminishes total blood cell count but also undermines cell viability, ultimately leading to DNA damage (16).

As a result, individuals residing in cold climates experience heightened stress-induced immune impairment compared to their counterparts in milder regions. Additionally, cold temperatures drive critical changes in cellular dynamics, particularly affecting Natural Killer (NK) cells—essential players in the innate immune response. The capacity of NK cells to target and eliminate infected or cancerous cells—referred to as NK cell activity (NKCA)—is a vital indicator of robust immune function. Moreover, NK cells are instrumental in orchestrating immune responses through the secretion of Type 1 and Type 2 cytokines. Thus, a decline in NK cell levels following exposure to cold temperatures significantly predisposes individuals to infections, underscoring the critical need for awareness and adaptation in cold environments (17). Cytokines are essential messenger molecules that orchestrate the inflammatory response. Produced by leukocytes, microglia, and astrocytes, these powerful substances play a critical role in the body’s defense mechanisms. Under normal circumstances, cytokines initiate complex signaling pathways that foster a balanced, protective immune response at the site of injury or infection (18). They can be classified into two categories: Type I cytokines, which amplify the inflammatory response (pro-inflammatory), and Type II cytokines, which mitigate it (19). However, when cytokines are overexpressed or their intricate equilibrium is disrupted, the inflammatory response can transform from a protective mechanism into a destructive force, leading to significant harm (20). Alarmingly, cold exposure also stimulates the production of myeloid-derived suppressor cells (MDSCs), which further stifle T cell proliferation, complicating the body’s immune response (14).

## 4 The role of antimicrobial extracellular vesicle (EV) swarms

Emerging evidence reveals that active mucosal defense against bacteria extends into the mucus itself, facilitated by the epithelial release of antimicrobial extracellular vesicle (EV) swarms (21). The study of extracellular vesicles (EVs) has experienced a remarkable transformation, fundamentally redefining our understanding of their role in both human health and the behavior of pathogenic microorganisms. Once dismissed as mere waste elimination tools, EVs are now recognized as essential players in cellular communication, driving critical processes in pathology and homeostasis. This shift in perspective underscores the importance of EVs in advancing our knowledge of cellular interactions and disease mechanisms (22).

EVs are remarkably small, membrane-bound particles released by cells that play a crucial role in intercellular communication. Their showcase remarkable diversity in shape, appearing ovoid, spherical, semilunar, or truncated, which facilitates a profound understanding of their biogenesis (23). These vesicles can be categorized into three primary types: exosomes, micro vesicles, and apoptotic vesicles (24).

The formation of EVs occurs through sophisticated mechanisms at specialized subcellular niches. Exosomes originate from the inward budding of late endosome membranes, resulting in intraluminal vesicles that are packaged within larger multivesicular bodies. These structures are then transported to and fused with the plasma membrane, releasing intraluminal vesicles as exosomes. In addition to exosomes, micro vesicles arise from plasma membrane blebbing, while other types, such as migrasomes, are formed from cytokinetic bridges or trails left by migrating cells. This complex array of manufacturing processes reflects the dynamic role EVs play in cellular communication and highlights their transformative potential as therapeutic agents (25). This vesiculation enables the export of diverse biomolecules within a single compartment, allowing for intricate interactions among different cells, even across unrelated organisms. Bacteria, leveraging this capability, can deploy vesiculation as either a synergistic or antagonistic communication system. The biomolecules contained within EVs exhibit varied properties that can be categorized into three compelling groups: identity properties, metabolic properties, and clinical/modulatory properties (26). These nanosized membranous entities are produced by nearly all cell types and play pivotal roles in intercellular communication. They facilitate the transfer of critical biomolecules—including proteins, lipids, DNA, RNA, and microRNA—between cells, thereby enabling the exchange of vital genetic information, signaling molecules, and cellular components. This intricate communication network is essential for various physiological processes, such as immune modulation, tissue regeneration, and neural communication (27). The specific composition of lipids, proteins, and nucleic acids within these vesicles fundamentally dictates their influence on target cells, facilitating interactions through receptor-ligand binding or through the transfer of molecular cargo. Importantly, the processes governing the incorporation of substances into EVs and their subsequent release are highly regulated and are intricately linked to both the type of cells involved and their physiological conditions. Understanding these mechanisms opens new avenues for harnessing EVs in therapeutic applications and biomedical research (28, 29). Moreover, EVs have emerged as key players in pathological conditions, including Alzheimer’s disease, various cancers, and cardiovascular diseases, underscoring their immense potential as both diagnostic markers and therapeutic agents (27). In the context of host responses to microbial infections, extracellular vesicles have emerged as critical players. Host EVs not only combat microbial invaders by targeting pathogen cells directly but also play a vital role in regulating immune responses (30, 31). Simultaneously, the responses instigated by microbial EVs can enhance immunity but may also precipitate disease symptoms (32).

Increasing research indicates that, in addition to their well-documented antibacterial properties, EVs may also play a pivotal role in regulating innate immune responses to viral infections (33). As pivotal agents of communication and modulation within the extracellular environment (34), EVs play a crucial role in the regulation of pathogenesis during both autologous and infectious processes (35). Notably, cells are constantly producing EVs, yet their composition is dynamically altered in response to stressful microenvironments, metabolic competition, varying phases of pathology, and detoxification processes (36).

A common thread that unites both animal and plant interactions is the recognition of microbe-associated molecular patterns (MAMPs) carried by microbial EVs. This recognition activates pattern recognition receptors (PRRs), triggering innate immune responses through mitogen-activated protein kinase (MAPK) pathways (30, 37). A mitogen-activated protein kinase (MAPK or MAP kinase) is a vital type of serine/threonine-specific protein kinase that plays a critical role in orchestrating cellular responses to a diverse range of stimuli, including mitogens, osmotic stress, heat shock, and pro-inflammatory cytokines. These powerful enzymes are essential in regulating key cellular functions such as proliferation, gene expression, differentiation, mitosis, cell survival, and apoptosis (38). By influencing these fundamental processes, MAPKs are integral to maintaining cellular health and resilience in the face of various environmental challenges. Significant differences characterize the functions of mammalian and plant EVs. Mammalian EVs are primarily engaged in modulating immune responses, particularly within the gut, while plant EVs serve a crucial role in transporting antimicrobial RNA molecules. However, as research in this field progresses, these distinctions may very well diminish, illustrating the complex interplay between EVs and immune systems across different biological realms (30).

A groundbreaking study conducted by Huang et al. (39) delves into the crucial function of extracellular vesicles (EVs) derived from the nasal epithelium in bolstering innate antiviral immunity through Toll-like receptor 3 (TLR3). Toll-like receptors (TLRs) are essential components of the innate immune response, serving as critical pattern recognition receptors that detect and defend against pathogens. These receptors are adept at identifying external pathogen-associated molecular patterns and are found on a wide range of innate immune cells, including macrophages, neutrophils, dendritic cells (DCs), natural killer (NK) cells, mast cells, basophils, and eosinophils. Upon activation, TLRs initiate powerful signaling cascades within the host, functioning not only as a robust defense mechanism against invading pathogens but also as agents of tissue repair. This dynamic activation results in the release of a diverse array of inflammatory cytokines and immune modulators, underscoring the pivotal role that TLRs play in safeguarding our health and orchestrating an effective immune response (40).

**Figure 1:**
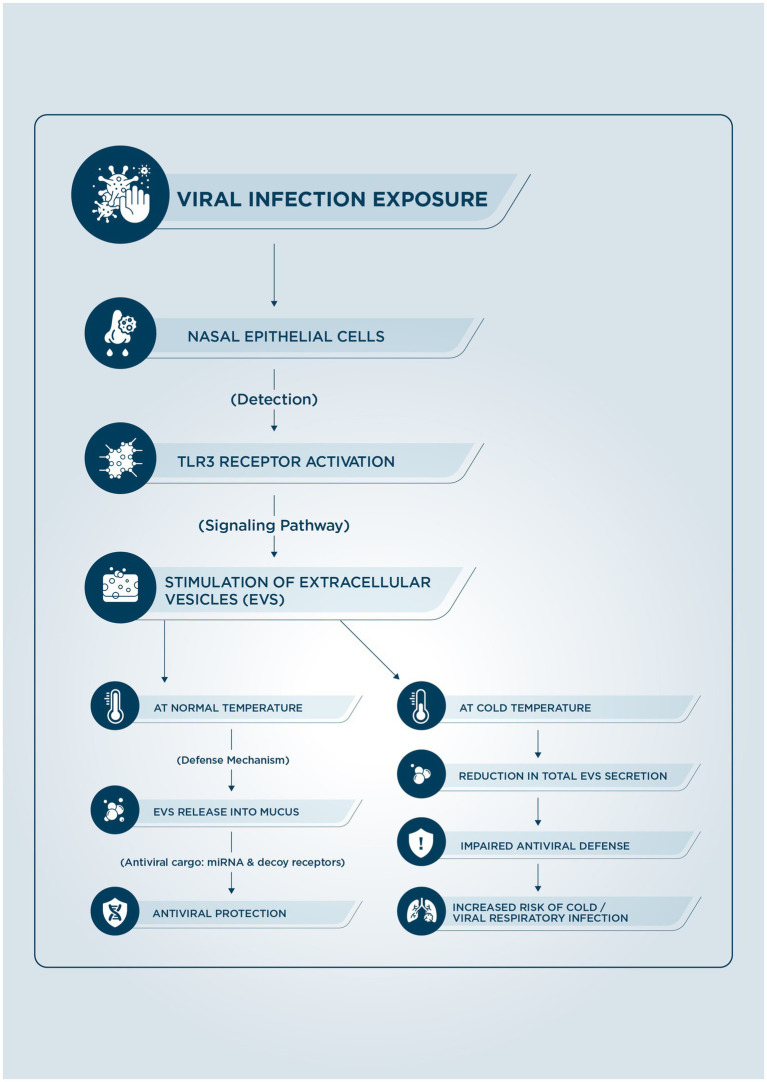
A diagram illustrating how cold temperatures impact the defense line of extracellular vesicles (EVs).

The researchers meticulously examined the secretion and composition of nasal epithelial EVs following TLR3 activation in both human autologous cells and fresh nasal mucosal surgical specimens. Additionally, they explored the potent antiviral effects of TLR3-stimulated EVs against respiratory viruses, as well as the detrimental impact of cool ambient temperatures on TLR3-dependent immunity. The results underscore that EVs are instrumental in achieving TLR3-mediated antiviral defenses, effectively safeguarding the host from viral infections. However, the study reveals a concerning finding: these robust antiviral mechanisms, facilitated by TLR3-stimulated EVs, are significantly weakened by exposure to cold environments. This impairment is evidenced by a reduction in total EV secretion, as well as diminished microRNA packaging and reduced antiviral binding affinity in individual EVs (Figure 1). These insights highlight the vital interplay between environmental factors and immune responses, emphasizing the need for further research in this area (39).

This evolving understanding underlines the importance of investigating EVs, as they hold promise for innovative therapeutic strategies against microbial infections and immune-related disorders.

## 5 Conclusion

Cold air can create a favorable environment for the proliferation of specific microorganisms. EVs are now recognized as essential players in cellular communication, driving critical processes in pathology and homeostasis. This evolving understanding underlines the importance of investigating EVs, as they hold promise for innovative therapeutic strategies against microbial infections and immune-related disorders. These insights emphasize the need for enhanced awareness and proactive strategies in managing URIs, especially during the colder months, to mitigate their impact on public health.

## Data Availability

The raw data supporting the conclusions of this article will be made available by the authors, without undue reservation.
